# Assessing exercise cardiac reserve using real-time cardiovascular magnetic resonance

**DOI:** 10.1186/s12968-017-0322-1

**Published:** 2017-01-23

**Authors:** Thu-Thao Le, Jennifer Ann Bryant, Alicia Er Ting, Pei Yi Ho, Boyang Su, Raymond Choon Chye Teo, Julian Siong-Jin Gan, Yiu-Cho Chung, Declan P. O’Regan, Stuart A. Cook, Calvin Woon-Loong Chin

**Affiliations:** 1 National Heart Centre Singapore, 5 Hospital Drive, Singapore, 169609 Singapore; 2 Changi General Hospital, Singapore, Singapore; 3 Siemens Healthineers, Erlangen, Germany; 4 MRC London Institute of Medical Sciences, London, UK; 5 Duke-NUS Medical School, Singapore, Singapore; 6 National Heart and Lung Institute, Imperial College, London, UK

**Keywords:** Cardiovascular magnetic resonance, Supine bike ergometer, Exercise physiology, Cardiopulmonary exercise test

## Abstract

**Background:**

Exercise cardiovascular magnetic resonance (ExCMR) has great potential for clinical use but its development has been limited by a lack of compatible equipment and robust real-time imaging techniques. We developed an exCMR protocol using an in-scanner cycle ergometer and assessed its performance in differentiating athletes from non-athletes.

**Methods:**

Free-breathing real-time CMR (1.5T Aera, Siemens) was performed in 11 athletes (5 males; median age 29 [IQR: 28–39] years) and 16 age- and sex-matched healthy volunteers (7 males; median age 26 [interquartile range (IQR): 25–33] years). All participants underwent an in-scanner exercise protocol on a CMR compatible cycle ergometer (Lode BV, the Netherlands), with an initial workload of 25W followed by 25W-increment every minute. In 20 individuals, exercise capacity was also evaluated by cardiopulmonary exercise test (CPET). Scan-rescan reproducibility was assessed in 10 individuals, at least 7 days apart.

**Results:**

The exCMR protocol demonstrated excellent scan-rescan (cardiac index (CI): 0.2 ± 0.5L/min/m^2^) and inter-observer (ventricular volumes: 1.2 ± 5.3mL) reproducibility. CI derived from exCMR and CPET had excellent correlation (*r* = 0.83, *p* < 0.001) and agreement (1.7 ± 1.8L**/**min/m^2^). Despite similar values at rest (*P* = 0.87), athletes had increased exercise CI compared to healthy individuals (at peak exercise: 12.2 [IQR: 10.2–13.5] L/min/m^2^ versus 8.9 [IQR: 7.5–10.1] L/min/m^2^, respectively; *P* < 0.001). Peak exercise CI, where image acquisition lasted 13–17 s, outperformed that at rest (c-statistics = 0.95 [95% confidence interval: 0.87–1.00] versus 0.48 [95% confidence interval: 0.23–0.72], respectively; *P* < 0.0001 for comparison) in differentiating athletes from healthy volunteers; and had similar performance as VO_2max_ (c-statistics = 0.84 [95% confidence interval = 0.62–1.00]; *P* = 0.29 for comparison).

**Conclusions:**

We have developed a novel in-scanner exCMR protocol using real-time CMR that is highly reproducible. It may now be developed for clinical use for physiological studies of the heart and circulation.

**Electronic supplementary material:**

The online version of this article (doi:10.1186/s12968-017-0322-1) contains supplementary material, which is available to authorized users.

## Background

Cardiac exercise testing is commonly used to detect underlying cardiovascular abnormalities that are not apparent at rest. Cardiovascular magnetic resonance (CMR) provides accurate assessment of cardiac volumes and function with excellent reproducibility compared to other standard imaging modalities [1, 2] but its application in stress testing has been limited to pharmacological agents.

Up until recently, the lack of suitable CMR-compatible exercise equipment and real-time imaging techniques have precluded accurate cardiac assessment of exercise physiology. Early studies in exercise CMR (exCMR) were performed using either breath-hold procedures that are not physiological or long free-breathing image acquisitions [3–5]. Although improvements in CMR technology have shortened the duration of exCMR imaging, these studies used a CMR-compatible treadmill [6–8]. The strengths of using treadmill exCMR are its validated diagnostic [9] and prognostic value and the ability to perform 12-lead electrocardiogram during exercise stress (the Duke score that also carries prognostic value) [10, 11]. However, a major limitation of performing treadmill exCMR is the obvious time delay needed to transfer the patient from the treadmill into the scanner.

In contrast, a supine cycle ergometer attached to the scan table will allow patients to exercise while in the bore. There are many advantages for exCMR particularly as images are acquired during the intermediate stages as well as at peak exercise, thus providing a large added value for statistical analyses of quantitative indices (e.g. stroke volume) and for repeated appreciation of qualitative changes (e.g. wall motion). However, excessive motion during exercise poses a challenge in image acquisition. Ungated real-time cine imaging and retrospective synchronisation of respiratory cycles have been used [5]. However, this translated to increased image acquisition time and complex image post-processing. We propose an approach of acquiring cine images at every stage of exercise during a brief period of exercise cessation to reduce artefacts from excessive motion and ECG-gating.

Using a CMR-compatible cycle ergometer and real-time CMR, we aimed to evaluate the feasibility and reproducibility of our exercise protocol; and to examine its potential to differentiate athletes from healthy volunteers.

## Methods

### Study population

A total of 11 athletes and 16 age- and sex-matched healthy volunteers were recruited to the study. Healthy volunteers did not have any diagnosed cardiac conditions and cardiovascular risk factors (hypertension, diabetes mellitus and hyperlipidemia). All the athletes competed in national/international events and trained more than 10 h a week in a variety of sports: triathlons (*n* = 7), long distance running (*n* = 1), rowing (*n* = 1), rugby (*n* = 1) and badminton (*n* = 1). We used the well-validated General Practice Physical Activity Questionnaire to assess physical activity levels in all participants, and classified them into four categories: inactive, moderately inactive, moderately active and active [12]. A five-point score was used to assess participant’s experience during exCMR (1 = would not do it again to 5 = highly satisfied; 3 = neutral).

The study was conducted in accordance with the Declaration of Helsinki and approved by the Singhealth Centralised Institutional Review Board. Written informed consent was obtained from all individuals.

### Exercise CMR protocol

**Fig. 1:**
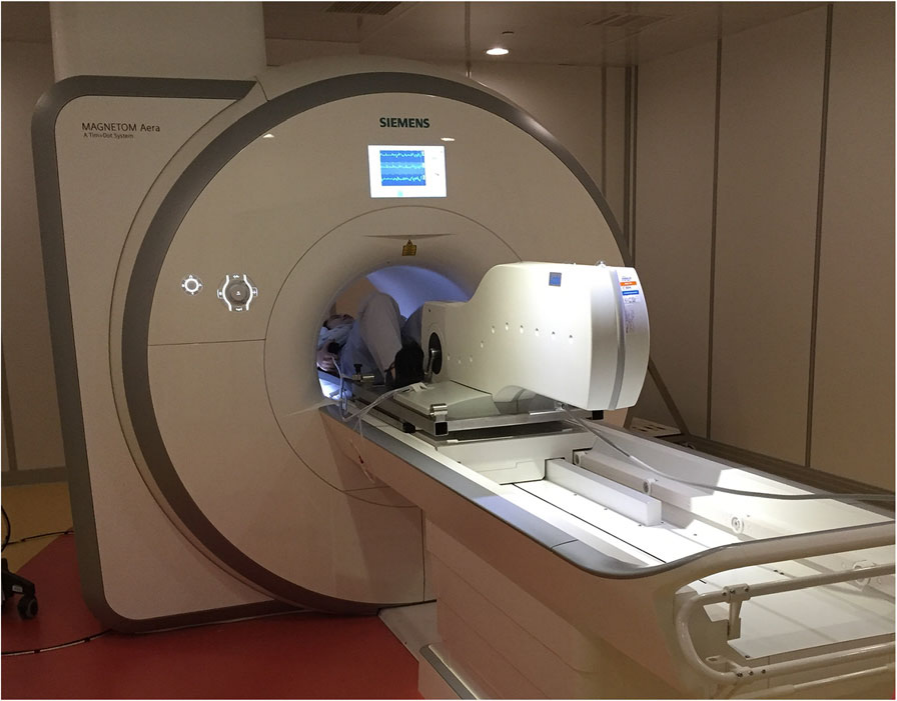
Set up of the cycle ergometer in CMR scanner

**Fig. 2:**
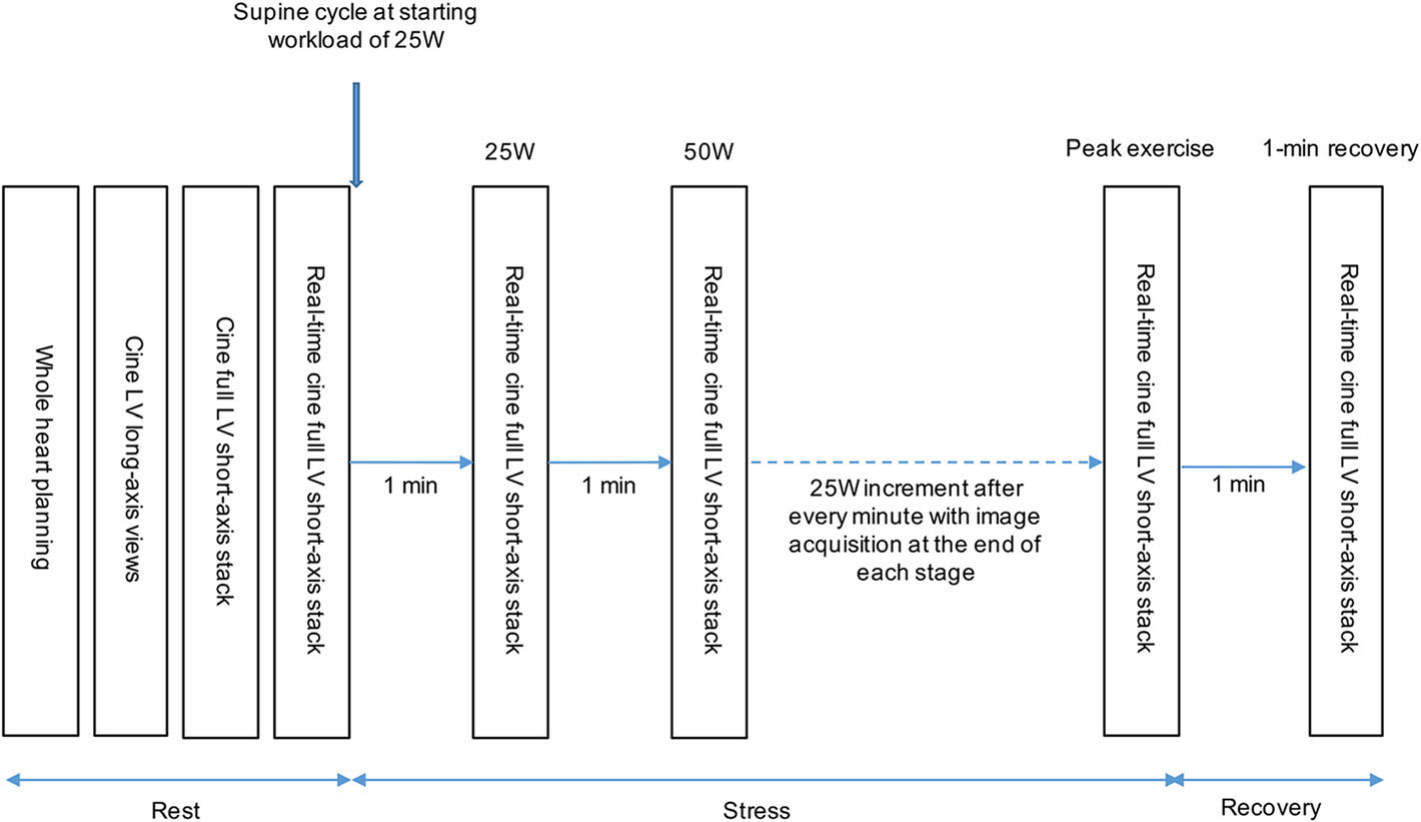
Exercise CMR imaging protocol

Exercise was performed using a programmable supine ergometer (Lode BV, Netherlands) fitted onto the CMR scanner table (1.5T MAGNETOM Aera, Siemens, Erlangen, Germany) (Figs. 1 and 2). Images were acquired using the 60-channel cardiac coils (30 anterior and 30 posterior elements).

After obtaining the baseline images, the participants were asked to cycle at an initial workload of 25W, with cadence maintained at least 70 rpm for 1 min. Workload was increased by 25W every minute until exhaustion. Free-breathing imaging was performed at the end of every stage during a brief period of stopping exercise. This is to avoid poor ECG signal and excessive motion artefacts that might result during exercise. Blood pressure and heart rate were recorded at every stage of exercise.

The imaging sequences and parameters were as follows:

- Balanced steady-state free precession (bSSFP): standard long axis (vertical, horizontal long axis, sagittal LV outflow tract) and short axis cines (extending from the base to apex) were performed in all patients before initiating exercise (8mm thick and 2mm gap; TE 1.2 ms; TR 3 ms; 280–320 mm field of view; 13 segments per phase; acquired matrix size 205 × 256 pixels; acceleration factor of 2; acquired voxel size 1.6 × 1.3 × 8.0 mm; 30 phases per cardiac cycle).
- Real-time bSSFP short axis cines: prospective ECG-gated free-breathing image acquisition of 10–13 short axis cine slices, extending from the base to apex was performed at each stage of the exercise (8 mm thick and 2 mm gap; TE 0.99 ms; TR 2.3 ms; 225 × 300 mm field of view; phase FOV 75%; acquired matrix size 68 × 128 pixels; phase resolution 71%; acceleration factor of 4; acquired voxel size 3.3 × 2.3 × 8.0 mm), temporal resolution 39.1 ms. Two different acquisition duration per slice were used in image acquisition: 1500 ms for heart rate less than 80 bpm (scan duration between 17 and 22 s); and 1200 ms for heart rate greater than 80 bpm (scan duration between 13 and 17 s). The rationale of using different acquisition durations was to obtain sufficient frames per cardiac cycle to capture end-diastole and end-systole at the different exercise stages. Using this approach, we were able to capture at least 2 cardiac cycles per exercise stage (Additional file 1: Video S1, Additional file 2: Video S2 and Additional files 3: Video S3).
Additional file 1: Video S1.Breath-hold cine images at baseline. (MOV 857 KB)
Additional file 2: Video S2.Real-time cine images at baseline. (MOV 1010 KB)
Additional file 3: Video S3.Real-time cine images at peak exercise. (MOV 780 KB)

### Image analysis

**Fig. 3:**
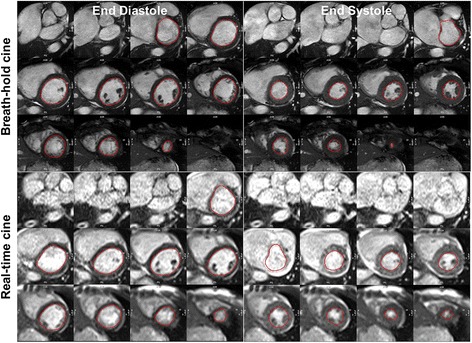
Endocardial contours for volume measurements of breath-hold cine and real-time cine images

Left ventricular (LV) endocardial borders in short axis cine images, at end diastole and end systole, were manually contoured at baseline and at each exercise stage (CVI42, Circle Cardiovascular Imaging Inc., Canada). Stroke volume (SV) was measured as the difference between maximum volume (end-diastolic volume, EDV) and minimum volume (end-systolic volume, ESV) across the cardiac cycles (Fig. 3). Additional steps to manually select and compose the end-diastolic and end-systolic phases may be required if there was misalignment of phases due to ECG mis-triggering. Cardiac output (CO) was calculated as: CO = SV x HR. All measured volumes and cardiac output were indexed to body surface area (DuBois formula).

### Reproducibility and validation

In 10 individuals, a repeat scan using the same exercise protocol was performed at least 7 days from the first scan to assess scan-rescan reproducibility.

A total of 20 individuals (healthy volunteers, *n*=10 and athletes, *n*=10) underwent additional cardiopulmonary exercise test (CPET), the gold standard of assessing exercise capacity. CPET was performed on an upright cycle ergometer (Vmax Encore E229C, USA) with a similar incremental protocol beginning at 50W and 25W increments every minute until exhaustion. Oxygen consumption at baseline (VO_2_) and at peak exercise (VO_2max_) were measured (Viasys Healthcare Cardiosoft, version 20). Cardiac output can be estimated from VO_2_ as: $\mathrm{C}\mathrm{O}=\frac{{100\times \mathrm{V}\mathrm{O}}_2}{5.721+\left(0.1047\times \%{\mathrm{VO}}_{2 \max}\right)}$ [13].

### Statistical analysis

All continuous variables were assessed for normal distribution and presented as mean±standard deviation or median [interquartile range (IQR)], as appropriate. Heart rate was normalized to the percent change between rest (0%) and peak heart rate (100%).

Pearson’s correlation and linear regression were used to assess the association between exercise capacities measured from exCMR and from CPET. Fixed and proportional biases with 95% limits of agreement between these two techniques were assessed using the Bland-Altman analysis. The performance of exCMR was assessed using the *c* statistics for discrimination [area under the receiver operating curve].

All statistical analyses were performed using GraphPad Prism 7 (GraphPad Software, Inc., San Diego, CA) and SPSS version 24 (IBM Corp., Armonk, NY). A 2-sided *P* <0.05 was considered statistically significant.

## Results

### Baseline characteristics

Athletes had larger cardiac volumes and increased LV mass compared to healthy individuals (*P*<0.05 for all comparisons). In athletes and healthy individuals, there were no sex-related differences in indexed SV and cardiac index (CI) (P>0.05 for all comparisons, Table 1). All athletes exercised more than 10 h a week and considered active based on the questionnaire. On average, healthy volunteers exercised between 1 and 3 h a week and were considered moderately active based on the questionnaire.

**Table 1:** Baseline characteristics and ventricular measurements

Parameters at baseline	Healthy volunteers (*n* = 16)	Athletes (*n* = 11)	*P* value
Clinical variables			
Age, years	26 [25–33]	29 [28–39]	0.121
Males, n (%)	7 (44)	5 (45)	0.93
Systolic blood pressure, mmHg	117 [107–123]	119 [107–127]	0.815
Diastolic blood pressure, mmHg	67 [59–75]	68 [66–74]	0.446
Heart rate, beats per minute	68 [59–74]	54 [48–61]	0.026
Body surface area, m^2^	1.64 [1.51–1.96]	1.64 [1.50–1.97]	0.394
Cardiovascular variables			
LV mass, g	76 [65–97]	100 [93–122]	0.008
LV end-diastolic volume, mL	141 [115–169]	169 [145–188]	0.121
LV end-systolic volume, mL	54 [43–79]	79 [62–83]	0.05
LV stroke volume, mL	88 [74–104]	101 [80–104]	0.422
Cardiac output, L/min	5.8 [4.5–6.3]	5.6 [4.0–6.6]	0.610
RV end-diastolic volume, mL	150 [121–197]	189 [154–208]	0.178
RV end-systolic volume, mL	69 [45–99]	90 [74–100]	0.231
Indexed LV mass, g/m^2^	47 [40–53]	66 [6–74]	<0.001
Indexed LV end-diastolic volume, mL/m^2^	82 [75–94]	102 [96–110]	<0.001
Indexed LV end-systolic volume, mL/m^2^	33 [28–40]	48 [43–49]	0.001
Indexed LV stroke volume, mL/m^2^	52 [48–54]	56 [54–63]	0.003
Cardiac index, L/min/m^2^	3.3 [2.8–3.9]	3.4 [2.6–4.1]	0.865
Indexed RV end-diastolic volume, mL/m^2^	91 [81–107]	112 [106–122]	0.002
Indexed RV end-systolic volume, mL/m^2^	40 [30–53]	56 [45–59]	0.023

*Abbreviations: LV* left ventricle, *RV* right ventricle

### Exercise CMR protocol

All subjects exercised until they felt they could not continue. Median exercise duration was 7 [IQR: 7–10] minutes in healthy volunteers and 9 [IQR: 8–11] minutes in athletes. The duration of exercise cessation for image acquisition in between each stage ranged between 15 and 25 s, depending on the heart rate. As heart rate increased in the later stages of exercise, the cardiac cycle length became shorter and therefore, shorter acquisition times. The drop in heart rate from the end of exercise to the end of image acquisition was 9±6 bpm in healthy volunteers and 16±9 bpm in athletes. The exCMR was well-tolerated by all participants (median satisfaction score of 4 [IQR: 3–4]). At baseline, LV EDV and ESV measured from the baseline real-time and breath-hold cine images demonstrated excellent agreement (**Indexed EDV**: mean difference of 0.8±1.8 mL/m^2^; **Indexed ESV**: 0.4 ±1.9 mL/m^2^).

**Fig. 4:**
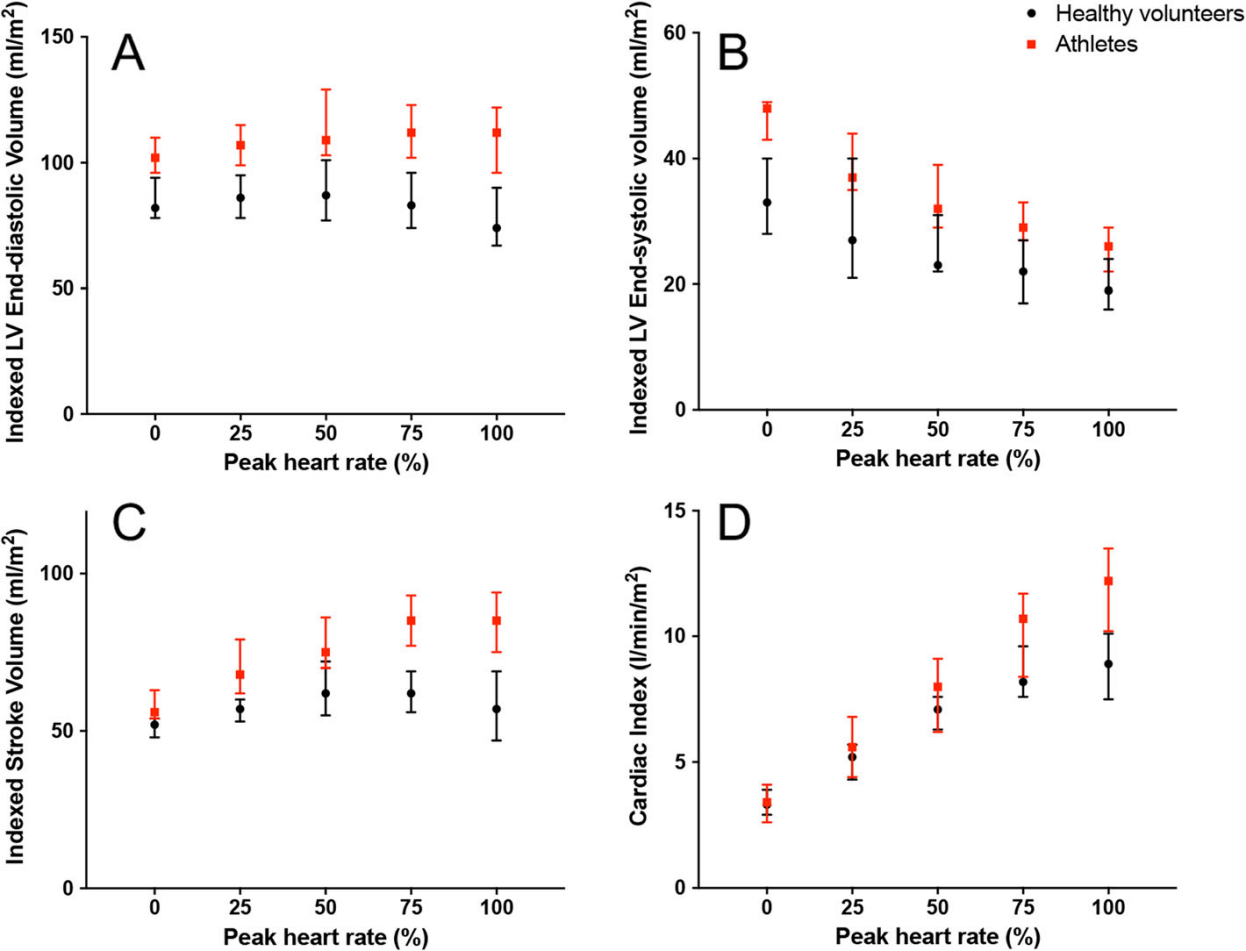
Exercise Cardiac Reserve in Athletes and Healthy Volunteers. Changes in indexed LV end-diastolic volume (**a**), indexed LV end-systolic volume (**b**), indexed stroke volume (**c**) and cardiac index (**d**) during exercise in healthy volunteers and athletes. Data presented in median (dots) and interquartile range (bars)

**Fig. 5:**
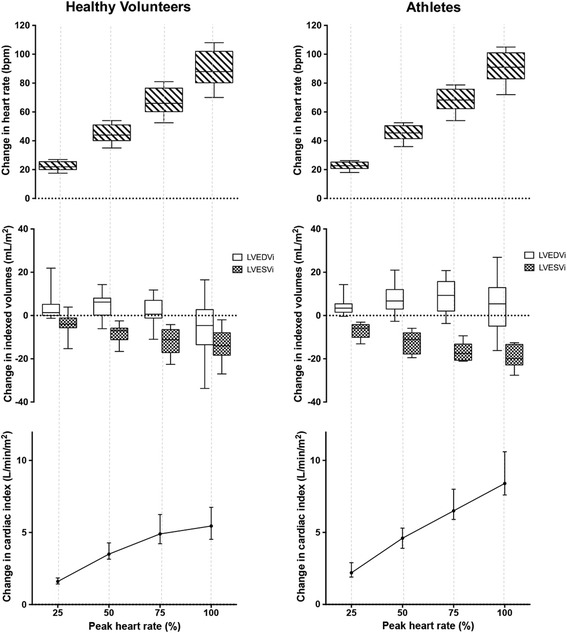
Cardiac Response to Exercise. Absolute Change in Cardiac Volumes From Baseline in Healthy Volunteers (*Left*) and Athletes (*Right*). Results are presented in box-and-whiskers plot (minimum and maximum); and median with interquartile range for line plot

In the intermediate stages of exercise, there were notable differences between athletes and healthy volunteers. In athletes, LV EDV increased at about 75% peak heart rate and remained elevated at peak exercise despite increasing heart rate; whilst in healthy volunteers, LV EDV peaked earlier (about 50% peak heart rate) and decreased subsequently (Fig. 4). LV ESV decreased at every stage of exercise in athletes and healthy volunteers (Figs. 4 and 5). This accounted for the different SV profiles: indexed SV peaked at 75% peak heart rates in both athletes and healthy volunteers. Whilst indexed SV remained elevated at peak exercise in athletes, it decreased in healthy volunteers (Fig. 4). The chronotropic response in the later stages of exercise differed between athletes and less fit healthy volunteers: increased heart rate augment CI in athletes, but not in healthy volunteers (Fig. 4).

At peak exercise, heart rate increased by 238±39% and 264±32% in healthy volunteers and athletes, respectively. This corresponded to 83±6% and 78±7% of age-predicted maximal heart rate in healthy individuals and athletes, respectively. Despite similar CI at rest (3.3 [IQR:2.8–3.9] L/min/m^2^ versus 3.4 [IQR: 2.6–4.1] L/min/m^2^; *P*=0.87), athletes had increased CI compared to healthy volunteers at peak exercise (12.2 [IQR: 10.2–13.5] L/min/m^2^ versus 8.9 [IQR: 7.5–10.1] L/min/m^2^; *P*<0.001; Table 2). Similar to baseline values, there were no sex-related differences in CI at peak exercise in athletes and healthy volunteers (P>0.05 for all comparisons). Therefore, we combined both sexes in the subsequent analysis. Unlike at rest, CI at peak exercise demonstrated excellent ability in differentiating athletes from healthy volunteers (c-statistics=0.48 [95% confidence interval: 0.23–0.72] versus 0.95 [95% confidence interval: 0.87 to 1.00]; *P*<0.0001 for comparison).

**Table 2:** Peak exercise comparison between healthy volunteers and athletes

Parameters at peak exercise	Healthy volunteers (*n* = 16)	Athletes (*n* = 11)	*P* value
Clinical variables			
Heart rate, bpm	156 [150–165]	145 [135–158]	0.071
Systolic blood pressure, mmHg	128 [103–192]	150 [120–165]	0.640
Diastolic blood pressure, mmHg	90 [59–134]	68 [40–109]	0.379
Maximum exercise power, W	125 [107–150]	200 [175–225]	0.011
Cardiovascular variables			
LV end-diastolic volume, mL	135 [104–152]	171 [150-208]	0.005
LV end-systolic volume, mL	32 [26–47]	42 [36–46]	0.178
LV stroke volume, mL	96 [73–121]	129 [115–158]	0.003
Cardiac output, L/min	15 [12–19]	20 [16–23]	0.023
Indexed LV end-diastolic volume, ml/m^2^	75 [67–90]	112 [96–122]	<0.001
Indexed LV end-systolic volume, ml/m^2^	20 [16–24]	26 [22–28]	0.044
Indexed stroke volume, ml/m^2^	57 [47–69]	85 [75–94]	<0.001
Cardiac index, L/min/m^2^	8.9 [7.5–10.1]	12.2 [10.2–13.5]	<0.001

*Abbreviations*: *LV* left ventricle, *RV* right ventricle

### Reproducibility of exCMR and comparison with CPET

**Fig. 6:**
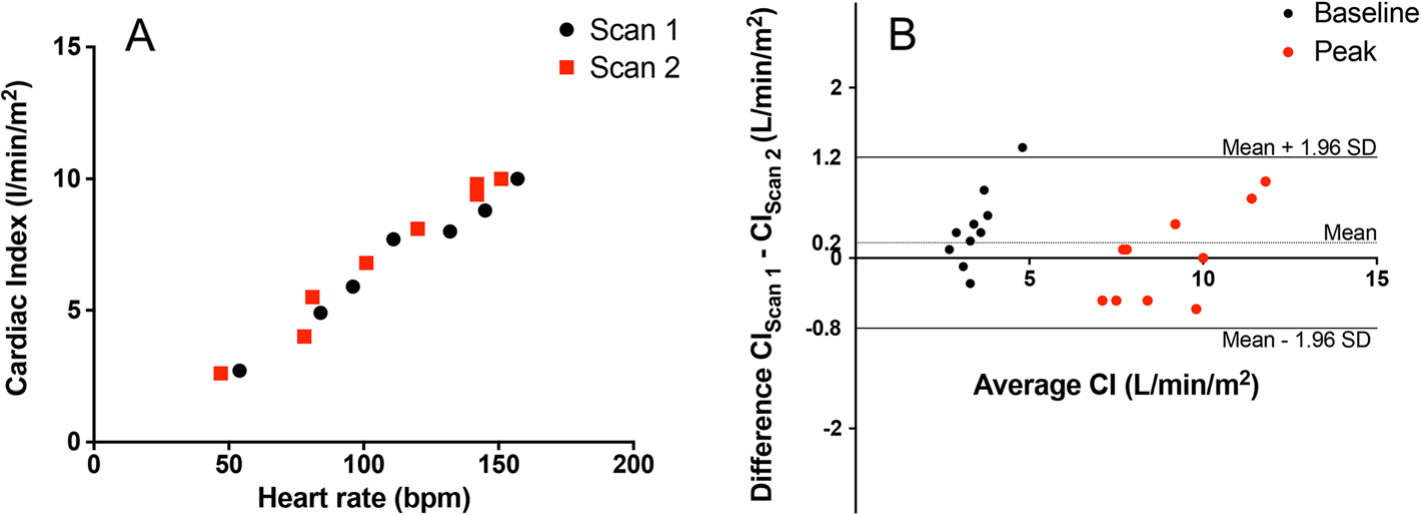
Scan-rescan reproducibility. Example of the exercise profile of an individual performed in the two scans (**a**); Bland-Altman plot of the difference in cardiac index measured between the two scans (**b**)

The exCMR protocol demonstrated excellent scan-rescan reproducibility with no difference in CI between the two scans performed at least 7 days apart (0.2±0.5 L/min/m^2^, Fig. 6). Moreover, we observed excellent inter-observer variability in the assessment of cardiac volumes (**LVEDV**: 2.8±5.2 mL; **LVESV**: –0.5±5.2 mL).

**Fig. 7:**
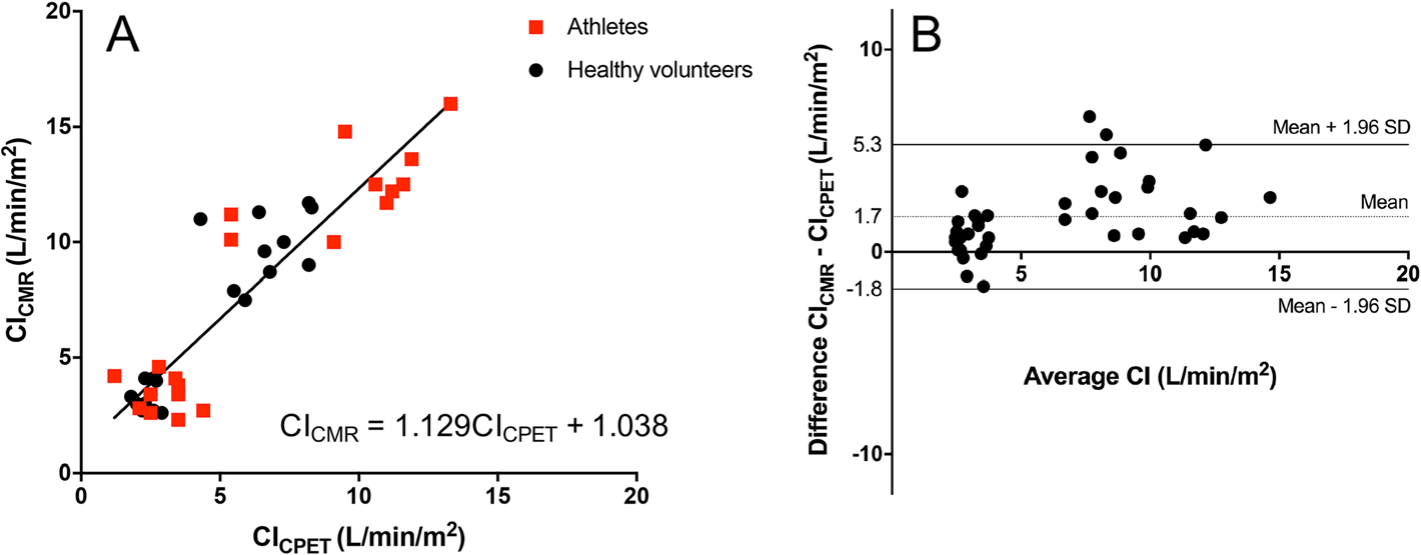
Correlation and Agreement in Cardiac Index Derived from Exercise CMR and CPET. Linear regression of cardiac index values measured from CMR and derived from CPET at rest and peak exercise (**a**) and Bland-Altman plot of the difference between cardiac index measured from CMR and estimated from CPET, at rest and peak exercise (**b**)

Of the 20 individuals who underwent both exercise tests, VO_2max_ was significantly higher in athletes compared to healthy individuals (50.7 [IQR: 39.4 to 56.9] mL/kg/min versus 29.8 [IQR: 28.3 to 34.0] mL/kg/min, respectively; *P*<0.001). Despite similar CI achieved (1.7±1.8 L**/**min/m^2^; Fig. 7), the maximal workload on the CMR supine ergometer was significantly lower compared to upright cycle CPET (**Athletes**: 200 [IQR: 175–225] W versus 237 [IQR: 213–300] W, *P*=0.007; **healthy volunteers**: 125 [IQR: 100–150] W versus 188 [IQR: 144–206] W, *P*=0.007). All participants exercised to the point they could not continue: the maximal exCMR workload was equivalent to the CPET workload at 80 [IQR: 70–86]% VO_2max_, much higher than the maximal workload defined at 60% VO_2max_ in other studies [3, 5]. The CI from exCMR correlated very well with both CPET-derived CI (*r*=0.83, *P*<0.001; Fig. 7) and CPET VO_2max_ (*r*=0.64, *P*=0.003). Compared to VO_2max_, exCMR-derived CI at peak exercise demonstrated similar ability to differentiate healthy volunteers from athletes (c-statistics=0.84 [95% confidence interval: 0.62–1.00] versus 0.95 [95% confidence interval: 0.87 to 1.00], respectively; *P*=0.292 for comparison).

## Discussion

We have developed an exCMR protocol with excellent inter-observer and scan-rescan reproducibility. We have also observed excellent correlations and agreement in exercise capacity between exCMR and the gold standard CPET. Using the exercise protocol, we were able to characterise exercise physiology at every stage; and demonstrated excellent ability in differentiating athletes from healthy volunteers (c-statistics=0.95 [95% CI: 0.87 to 1.00]; *P*<0.0001).

ExCMR requires appropriate exercise equipment, rapid and robust real-time image acquisition to accommodate free-breathing exercise protocols. A previous study tested the feasibility of treadmill placed outside the MR scanner room and used breath-hold imaging protocol [14]. This took 60–90 s to complete post-exercise imaging because of the time needed to transfer patients from the treadmill to the MR scanner and image acquisition. Subsequent studies adopted a modified treadmill in the MR scanner room and free-breathing acquisition protocols, with some improvement in post-exercise imaging time [6–8, 15]. Although the acquisition time was faster compared to previous studies, imaging was only carried out at maximal exercise and not at every stage (which is a potential strength in supine bike protocols).

The recent use of in-scanner cycle ergometer has eliminated any delay in transferring patients into the scanner after exercise, but excessive motion poses a challenge in image acquisition during exercise [5]. Ungated real-time cine imaging and retrospective synchronisation of respiratory cycles have been used to reduce motion, at the expense of increased image acquisition time and complex image post-processing [5]. Although phase-contrast imaging can reduce image acquisition time, it is not able to assess wall motion abnormalities in myocardial ischemia [4, 16, 17].

We demonstrated rapid acquisition of free-breathing peak-exercise cine images within 13 to 17 s and superior spatiotemporal resolution (spatial resolution: 3.3 × 2.3 mm; temporal resolution < 40ms). In our exercise protocol, we used two different acquisition duration (1500ms and 1200ms per slice for heart rates less than and more than 80, respectively) to ensure at least 2 cardiac cycles that would adequately identify the end diastolic and systolic phases. Importantly, the faster acquisition did not compromise image quality. Moreover, the increased number of coil elements may reduce artefacts that may affect the accurate assessment of cardiac volumes and function; and wall motion abnormalities. Indeed, we were able to achieve excellent scan-rescan and inter-observer variability; and did not observe any difference in ventricular volumes when compared using standard breath-hold and our free-breathing protocols.

CPET is widely accepted as the most reliable and objective test for assessing cardiac reserve in a variety of cardiac conditions, such as heart failure and distinguishing physiologic from pathologic left ventricular hypertrophy [18, 19]. We used similar exercise protocols for both the in-scanner cycle and upright cycle in CPET. All participants were given clear instructions to exercise until they were not able to continue anymore to ensure maximal exercise capacity was achieved. Of note, the maximum workload on the supine cycle ergometer was lower compared to upright cycle CPET. It is perhaps not surprising that the maximal workload would vary across different exercise stress modalities because of different cardiac responses. Despite different workload attained, supine and upright cycling demonstrated similar VO_2max_ [20, 21], but lower than treadmill CPET [11, 22]. Moreover, we observed excellent correlation and agreement in cardiac index between supine exCMR and upright cycle CPET, supporting the validity of the study.

During exCMR, we observed differences in exercise physiology between athletes and healthy individuals. The rate of increase of heart rate in response to exercise was similar in both groups (Fig. 5). Whilst athletes had augmented diastolic filling (increased LV EDV) and improved contractility (decreased LV ESV) throughout the range of exercise intensity tested, healthy volunteers attained peak diastolic filling and contractility earlier (at about 50% peak heart rate). At higher exercise intensity, increased chronotropic response further augment cardiac output in athletes but not in the less fit healthy volunteers. In healthy volunteers, the rapid heart rates reduced diastolic filling and therefore, stroke volumes at peak exercise. There are some uncertainties in the mechanisms associated with cardiac output augmentation in athletes and non-athletes [23–27]. Our study adds novelty by demonstrating mechanistic differences in exercise profiles between athletes and healthy volunteers using the same exercise protocol.

### Clinical implications

The study highlighted the potential of further extending the clinical applications of CMR to assess cardiac reserve. In addition to CMR being the gold standard for assessing left ventricular mass and cardiac volumes, it is the only imaging modality that can detect myocardial fibrosis non-invasively. The combination of these techniques in a single imaging modality offers valuable diagnostic insights in individuals with cardiac pathologies, which not achievable with CPET. The ability to assess cardiac physiology and function at every stage of exercise provides a unique opportunity to characterise and differentiate the exercise profiles between individuals. In this study, we have demonstrated one such potential application: peak exercise CI significantly outperformed CI at rest and had similar performance as VO_2max_ in differentiating athletes from healthy volunteers. This technique holds promise in distinguishing physiologic from pathologic myocardial biology in patients with LV dilatation, hypertrophy or mildly impaired systolic ejection fraction [28, 29]. The excellent reproducibility adds strength to use exCMR for serial assessments.

### Limitations

The CMR protocol required a brief period of stopping exercise at the end of every stage in order to minimise excessive motion and ECG artefacts during image acquisition. This resulted in a small drop in heart rate (9±6 bpm in controls and 16±9 bpm in athletes). However, the effect may be less significant in patients with cardiac pathologies or individuals who are older and less physically-active. The maximal heart rate achieved using this exCMR protocol was less than the recommended 85% age-predicted maximal heart rate (APMHR) commonly used in defining an adequate treadmill stress test [11, 30]. This may affect the diagnostic accuracy in assessing myocardial ischemia. However, it is well described that heart rates achieved with supine cycling is lower than exercise treadmill [22, 31, 32]; and previous echo studies have demonstrated similar diagnostic accuracies between supine and treadmill exercise stress, despite a large proportion of patients not achieving the 85% APMHR [31–33]. It is conceivable that supine exercise stress may not be suitable in some individuals (particularly those who are not accustomed to cycling) because of the postural effects on musculoskeletal fatigue.

## Conclusions

These data demonstrate the feasibility, accuracy and reproducibility of an in-scanner exercise protocol using a CMR-compatible cycle ergometer. Future studies will examine its clinical utility in a variety of cardiac pathologies.

## Abbreviations

APMHR: Age-predicted maximal heart rate
CI: Cardiac Index
CMR: Cardiovascular magnetic resonance
CO: Cardiac output
CPET: Cardiopulmonary exercise test
EDV: End-diastolic volume
ESV: End-systolic volume
exCMR: Exercise CMR
LV: Left ventricular/left ventricle
SV: Stroke volume
TGRAPPA: Temporal generalized auto-calibrating partially parallel acquisition
VO_2_: Oxygen consumption
VO_2max_: Oxygen consumption at peak exercise
